# Suppression of Cytochrome P450 Reductase Enhances Long-Term Hematopoietic Stem Cell Repopulation Efficiency in Mice

**DOI:** 10.1371/journal.pone.0069913

**Published:** 2013-07-26

**Authors:** Yan Zhang, Fang Dong, Na Zhang, Hui Cheng, Yakun Pang, Xiaomin Wang, Jing Xu, Xinxin Ding, Tao Cheng, Jun Gu, Weiping Yuan

**Affiliations:** 1 State Key Laboratory of Experimental Hematology, Institute of Hematology and Blood Disease Hospital, Chinese Academy of Medical Sciences & Peking Union Medical College, Tianjin, China; 2 Center for Stem Cell Medicine, Chinese Academy of Medical Sciences, Beijing, China; 3 Wadsworth Center, New York State Department of Health, and School of Public Health, SUNY at Albany, Albany, New York, United States of America; Emory University, United States of America

## Abstract

**Background:**

Bone marrow microenvironment (niche) plays essential roles in the fate of hematopoietic stem cells (HSCs). Intracellular and extracellular redox metabolic microenvironment is one of the critical factors for the maintenance of the niche. Cytochrome P450 reductase (CPR) is an obligate electron donor to all microsomal cytochrome P450 enzymes (P450 or CYP), and contributes to the redox metabolic process. However, its role in maintaining HSCs is unknown.

**Objective:**

To examine the effects of low CPR expression on HSCs function using a mouse model of globally suppressed Cpr gene expression (**C**pr **L**ow, CL mice).

**Methods:**

Hematopoietic cell subpopulations in bone marrow (BM) and peripheral blood (PB) from WT and CL mice were examined for their repopulation and differentiation ability upon BM competitive transplantation and enriched HSC (LKS^+^) transplantation. Effects of low CPR expression on hematopoiesis were examined by transplanting normal BM cells into CL recipients. Reactive oxygen species (ROS), cell cycle, and apoptosis in CL mice were analyzed by flow cytometry for DCF-DA fluorescence intensity, Ki67 protein, and Annexin-V, respectively.

**Results:**

The levels of ROS in BM cells, HPCs and HSCs were comparable between CL and WT mice. In comparison to WT mice, the number of LT-HSCs or ST-HSCs was lower in CL mice while CMPs, GMPs and MEPs in CL mice were higher than that in WT control. Competitive transplantation assay revealed enhanced repopulation capacity of HSCs with low CPR expression, but no difference in differentiation potential upon *in vitro* experiments. Furthermore, lymphoid differentiation of donor cells decreased while their myeloid differentiation increased under CL microenvironment although the overall level of donor hematopoietic repopulation was not significantly altered.

**Conclusions:**

Our studies demonstrate that suppressing CPR expression enhances the repopulation efficiency of HSCs and a low CPR expression microenvironment favors the differentiation of myeloid over lymphoid lineage cells.

## Introduction

The niche, and particularly its intracellular and extracellular redox metabolic microenvironment, is important for maintaining the self-renewal and differentiation of hematopoietic stem cells (HSCs) [1], [2]. Under normal condition, HSCs that possess long-term reconstitution ability, namely long term-HSCs (LT-HSCs), reside in amicroenvironment with low PO_2_ [3], [4], reportedly as low as 1% [5]. These HSCs express high level of Notch1, telomerase and p21 [6]. About 70% HSCs are in the G_0_ phase, with low cell metabolic activity [7]. The low levels of metabolism, cell cycling and ROS are required for maintaining self-renewal capability for HSC and the alteration in the levels of metabolism or the damage to HSC reduces the self-renewal ability of HSC and may thus result in HSC exhaustion [8], [9].

NADPH-cytochrome P450 oxidoreductase (CPR) is an obligated electron donor for all microsomal cytochrome P450 (P450s or CYP) enzymes [10]. P450s are responsible for metabolizing many foreign compounds as well as endogenous substances [11]. CPR and P450 are also involved in the production of ROS. CPR and P450 are expressed in almost all tissues, including the bone marrow cells. In the absence of the functional Cpr gene, P450 are catalytically inactive. Germline deletion of the Cpr gene causes embryonic lethality in mice [12]. In humans, *Cpr* mutation leads to congenital steroidogenesis deficiency, which in turn may result in Antley-Bixler syndrome, characterized by skeletal malformation and reproductive defects [13]. We propose that CPR/P450 system may also be critical for hematopoiesis. In the current study, we used a genetically engineered mouse model with only 5%–24% CPR expression in various tissues (CL mice) [14] to examine the roles of CPR/P450 system in HSC hematopoiesis. Specifically, we compared the CL mice with WT mice for their hematopoietic cell populations in the BM and PB, as well as the ability of HSCs for repopulation and differentiation using BM competitive transplantation and enriched HSC (LKS^+^) transplantation experiments. The impact of low CPR expression environment on hematopoiesis was examined by transplanting normal BM cells into CL recipients. The levels of ROS, cell cycle status, and apoptosis in the BM were also compared between the CL and WT mice.

## Materials and Methods

### Mice

C57BL/6J and B6.SJL were purchased from Vital River Laboratories (VRL, Beijing, China). The CL mice were generated and kindly provided by Dr. Xinxin Ding, Wadsworth Center, New York State Department of Health Albany, New York [14]. Briefly, the *Cpr* gene was disrupted by insertion of a *neo* gene in the intron 15 of the *Cpr* in CL mice, which led to a 74 to 95% decrease in CPR expression in all tissues examined, including olfactory mucosa, adrenal gland, brain, testis, ovary, lung, kidney, liver and heart. All mouse experiments were performed at the Institute of Hematology (IH), Tianjin, China. The mice used in the experiments have been backcrossed at least 10 times to the C57BL/6 background. If not specifically mentioned, sex matched WT and CL mice at 8–12 week-old were used in all the experiments. All mice were housed in individually ventilated micro-isolator cages in the same room of certified SPF grade animal facility at IH. The experimental protocol was approved by the Institutional Animal Care and Use Committee (IACUC), Institute of Hematology and Blood Disease Hospital, CAMS/PUMC.

### Antibodies for Flow Cytometry

Antibodies against CD34 (Clone: RAM34), FLK2/FLT32 (Clone: A2F10.1), c-Kit (Clone: 2B8), Sca-1 (Clone: D7), FcγRII/III (Clone: 93), CD3 (Clone: 145-2C11), B220 (Clone: RA3-6B2), CD11b (Mac-1) (Clone: M1/70), Gr-1 (Clone: RB6-8C5), CD4 (Clone: GK1.5), CD8 (Clone: 53-6.7), Ter-119 (Clone: TER119), IL-7Rα (CD127) (Clone: A7R34), CD45.1 (Clone: A20), and CD45.2 (Clone: 104) were from eBioscience (San Diego, CA, USA). The lineage cocktail contained Ter119, Gr-1, Mac-1, B220, CD3, CD4 and CD8 antibodies.

### Phenotypic Analysis

Subpopulation surface markers included Lin^−^c-kit^+^sca-1^+^Flk2^−^CD34^−^ for long-term hematopoietic stem cells (LT-HSC), Lin^−^c-kit^+^sca-1^+^Flk2^−^CD34^+^ for short-term hematopoietic stem cells (ST-HSC), Lin^−^c-kit^+^sca-1^+^Flk2^+^CD34^+^ for multi-potent progenitor cells (MPP), Lin^−^c-kit^+^sca-1^−^FcγRII/III^low^CD34^+^ for common myeloid progenitors (CMP), Lin^−^c-kit^+^sca-1^−^ FcγRII/III^−^CD34^−^ for megakaryocyte and erythrocyte progenitors (MEP), Lin^−^c-kit^+^sca-1^−^FcγRII/III^+^CD34^+^ for granulocyte and macrophage progenitors (GMP), Lin^−^IL-7R^+^c-kit^low^sca-1^low^ for common lymphoma progenitors (CLP), CD3^+^ for T lymphocytes B220^+^ for B lymphocytes, Mac-1^+^ for myeloid cells, Mac-1^+^Gr-1^+^ for granulocytes, and Lin^−^c-kit^+^sca-1^+^ for LKS^+^ cells. Samples were analyzed by LSR II or Arial II (BD, San Diego, CA, USA). All data were analyzed by Flowjo software (Treestar, Ashland, OR, USA).

### Enriched LKS^+^ Cell Competitive Transplantation

Bone marrow cells were harvested from mice by flushing bone marrow from tibias and femurs using 1 ml syringe connected with a 25GA needle. They were then gently passed through the needle to make a single-cell suspension. HSCs/HPCs were enriched using lineage cell depletion beads (Miltenyi, Germany). Enriched HSCs/HPCs were incubated with PE-Sca-1, APC-c-kit and PE/CY7-Lineage (eBioscience or BD Bioscience) and sorted using an Aria II sorter (BD, San Diego, CA, USA). LKS^+^ cells (2.5×10^3^) sorted either from C57BL/6J or CL mice plus CD45.1 WBMCs (whole bone marrow cells; 5×10^5^) from B6.SJL were transplanted into lethally irradiated (9.5 Gy) B6.SJL mice. PB was collected from the mice tail and analyzed every 4 weeks and bone marrow cells were analyzed 24 weeks after transplantation.

### Bone Marrow Cells Serial Transplantation

The bone marrow cells were isolated with the same method described above. CD45.2 whole bone marrow cells (WBMCs, 5×10^5^) from C57BL/6J or CL mice and CD45.1 WBMCs (5×10^5^) from B6.SJL were transplanted into lethally irradiated (9.5 Gy) B6.SJL mice, respectively. For 2^nd^ transplantation, CD45.2 cells (5×10^5^) sorted from 1^st^ transplantation recipient mice at 16 weeks post-transplantation and 5×10^5^ CD45.1 WBMCs (5×10^5^) from B6.SJL were transplanted into lethally irradiated (9.5 Gy) B6.SJL mice. For 3^rd^ transplantation, CD45.2 cells (2×10^5^) sorted from 2^nd^ transplantation recipient mice at 16 weeks post-transplantation plus CD45.1 WBMCs (2×10^5^) from B6.SJL were transplanted into lethally irradiated (9.5 Gy) B6.SJL mice.

### Recipient Transplantation

CD45.1 WBMCs (1×10^6^) from B6.SJL were transplanted into lethally irradiated C57BL/6J or CL mice. PB was analyzed every 4 weeks and BM cells were analyzed 24 weeks after transplantation.

### Western Blotting Analysis

Total protein of whole BM cells or sorted LKS^+^ cells from C57BL/6J or CL mice were obtained after RBCs were removed by lysis. Protein concentration was determined by BCA method (Thermo Scientific, USA). Samples (10 µg protein) were separated using a 10% SDS-PAGE gel, and transferred to nitrocellulose membranes. Membranes were blocked with 5% Nonfat milk for 1 h at RT and then incubated with CPR antibody (Abcam, Cambridge, England) or GAPDH antibody (Cell Signaling Technology, Beverly, MA, USA) at 4°C overnight. After washing with TBST, the membranes were incubated with a HRP-linked anti-rabbit IgG, antibody (Cell Signaling Technology) for 1 h at RT. Proteins of interest were visualized with ECL Plus (Amersham, NJ, USA) and quantified by Quantity One software (Bio-Rad, Hercules, CA, USA).

### Cell Morphology Studies

BM cells isolated from C57BL/6J or CL mice were stained with Wright-Giemsa staining, washed with fresh water, and dried for analysis.

### Colony Forming Assay

WBMCs (1×10^4^) isolated from C57BL/6J or CL mice were cultured in M3434 cytokine-enriched methylcellulose plates (Stem Cell Technologies, Vancouver, BC, Canada) for colony counting after 14 days.

### Cell Cycle Analysis

Isolated cells were stained with Ki67 (BD Pharmingen™, San Diego, CA, USA) and Hoechst33342 (Sigma, USA) for cell cycle analysis.

### Apoptosis Assay

Apoptosis was analyzed using PE Annexin-V Apoptosis Detection Kit (BD Pharmingen™, San Diego, CA, USA).

### Statistical Analyses

Continuous data were analyzed by two-tailed Student’s t-test (*P<0.05, **P<0.01, ***P<0.001). Enumeration data were analyzed by χ^2^ test.

## Results

### Low CPR Expression did not Affect ROS Levels in Bone Marrow Cells and HSCs in CL Mice

**Figure 1:**
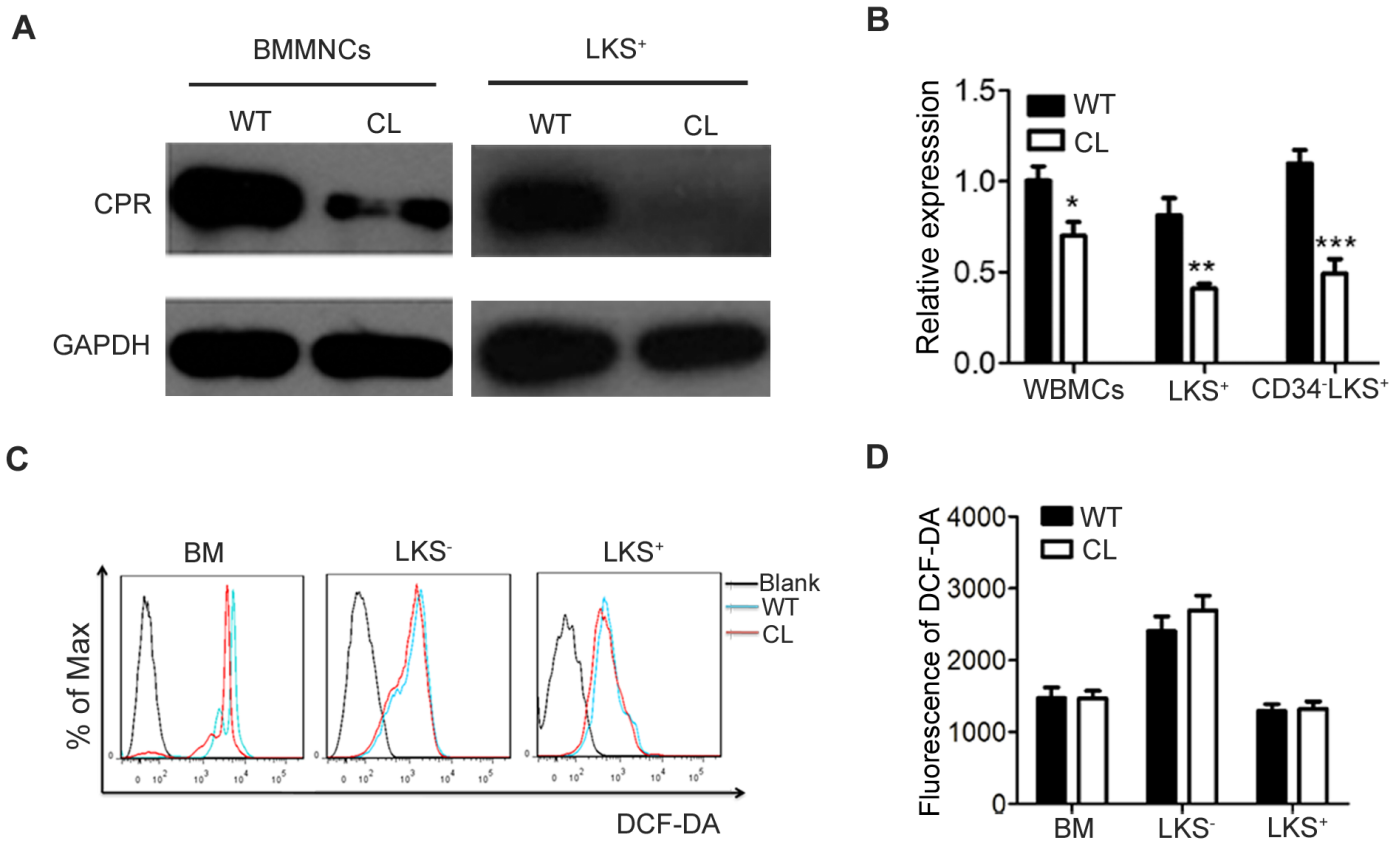
Similar ROS levels in BM cells between CL and WT mice. (**A**) CPR protein levels in bone marrow mononuclear cells (BMMNCs) and LKS^+^ of CL and WT mice were analyzed by Western blotting. (**B**) CPR mRNA expression in whole bone marrow cells (WBMCs), LKS^+^ and CD34^−^LKS^+^ were detected by qRT-PCR. Data shown are mean ± SEM (*P<0.05, **P<0.01, ***P<0.001, n3). (**C–D**) Representative FACS profiles (C) and quantification of the levels of ROS (D) in BM, LKS^−^ and LKS^+^ cells determined by DCF-DA mean fluorescence intensity with flow cytometry. Data shown are representative of two independent experiments.

Western blotting and qRT-PCR were used to determine CPR expression in bone marrow cells of CL mice at protein and mRNA levels. CPR in WBMCs and LKS^+^ cells of CL mice was decreased at protein level in comparison to that of WT mice (Figure 1A). Consistent with the results of Figure 1A, the mRNA expression of CPR in different subpopulations of bone marrow cells, including WBMCs, LKS^+^ and CD34^−^LKS^+^ of CL mice is much lower than that of WT mice (Figure 1B). The ROS levels in BM, LKS^−^ and LKS^+^ cell populations are comparable between CL and WT mice using DCF-DA mean fluorescence intensity analyzed by flow cytometry (Figure 1C and D). To evaluate the effect of low CPR expression on hematopoiesis, cell numbers in hematopoietic organs were quantified. WBC, RBC and PLT in PB and mononuclear cells in spleen, thymus and BM were also comparable between CL and WT mice (Figure S1A–C). These results indicated that CL mice maintain normal levels of ROS in BMCs although the expression of CPR is lower in the BMCs.

### CL Mice Showed Reduced HSC Frequency and Increased Myeloid Progenitor Frequency

**Figure 2:**
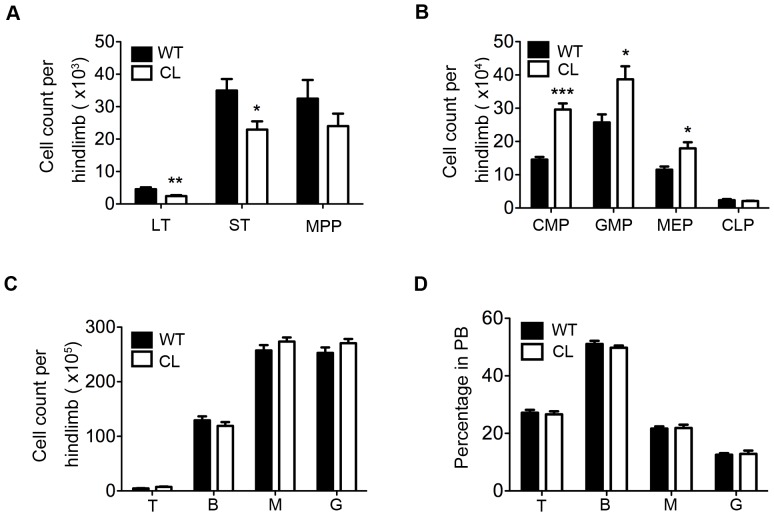
Decreased HSC frequency and increased myeloid progenitor frequency in CL mice. (**A–B**) Flow cytometry analysis of BM cells revealed decreased LT-HSCs and ST-HSCs and increased myeloid progenitors in CL mice in comparison with WT mice. Data shown are the mean ± SEM (*P<0.05, **P<0.01, ***P<0.001, n = 7–8). (**C–D**) T lymphocyte (T), B lymphocyte (B), myeloid cell (M) and granulocyte (G) in BM and peripheral blood (PB) in CL vs WT mice. Data shown are the mean ± SEM (n = 3–6).

In order to examine the effect of CPR on hematopoiesis, we analyzed hematopoietic sub-populations of CL mice by flow cytometry. The number of LT-HSCs and ST-HSCs was reduced in CL mice in comparison to that in the WT mice (Figure 2A). The number of CMP, GMP and MEP was increased in CL mice (Figure 2B). The number of mature cells, including T lymphocyte, B lymphocyte, myeloid cells and granulocytes in either BM or PB did not differ between the two strains (Figure 2C and D). The bone marrow cells of CL mice exhibited normal morphologies (Figure S2A and B) and displayed normal colony forming efficiency (Figure S2C).

### LKS^+^ Cells from CL Mice Have Enhanced Long-term Repopulation Potential

**Figure 3:**
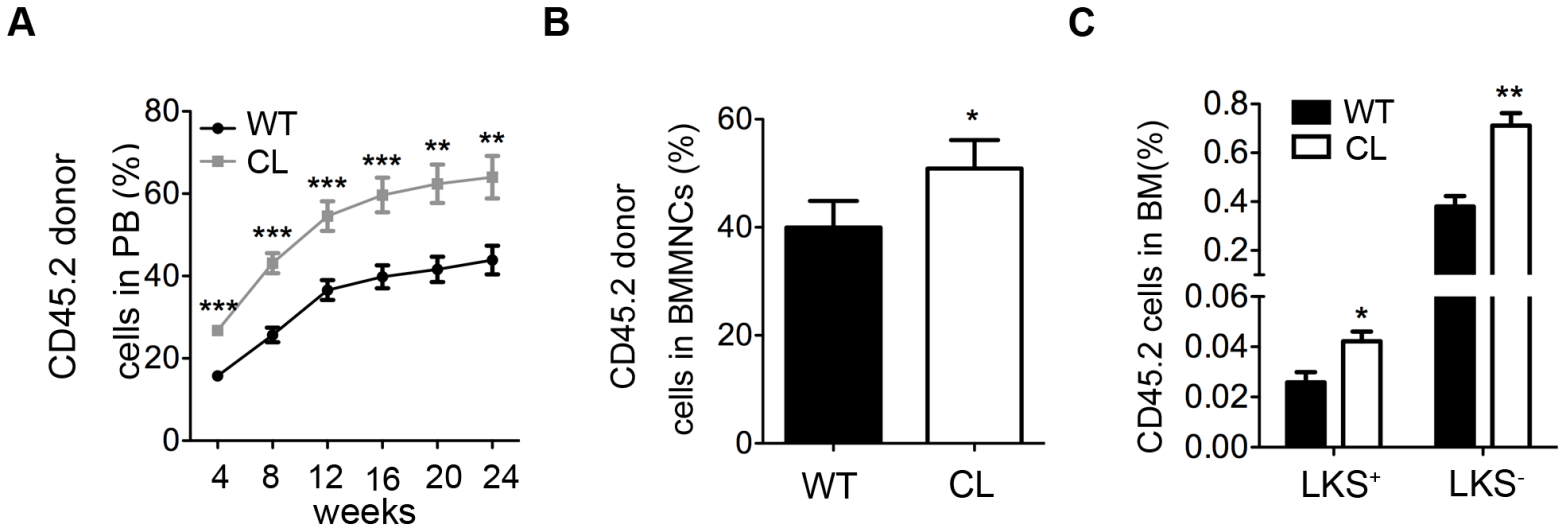
Enhanced long-term repopulation potential of LKS^+^ cells from CL mice. 2.5×10^3^ LKS^+^ cells from WT or CL mice plus 5×10^5^ CD45.1 WBMCs were transplanted into lethally irradiated (9.5 Gy) recipients (B6.SJL, CD45.1). (**A**) The CD45.2 donor cell percentage in PB was analyzed by flow cytometry every 4 weeks. Data shown are mean ± SEM (**P<0.01, ***P<0.001, n = 14–16). (**B**) The CD45.2 donor cell percentage in BM at 24 weeks after transplantation. Data shown are mean ± SEM (*P<0.05, n = 5). (**C**) The CD45.2 LKS cell percentage in BM at 24 weeks after transplantation. Data shown are mean ± SEM (*P<0.05, **P<0.01, n = 5).

Since the frequency of LT-HSCs and ST-HSCs were lower in CL mice, we set to investigate the effect of low CPR expression on HSC functions by the enriched LKS^+^ cell competitive transplantation experiment. We found that LKS^+^ of CL mice displayed enhanced long-term repopulation potential (Figure 3A). The percentage of CL donor cells (CD45.2^+^) in recipient mouse BM was higher at 24 weeks after transplantation in comparison to that in the WT donor cells (Figure 3B and C), indicating that low CPR expression enhances long-term HSC repopulation potentials. Interestingly, the increase was proportional for mature donor cells in PB and BM and thus no proportion change of composition in different cell lineages between CL and WT mice (S3A-D). This suggests that the differentiation abilities of LKS^+^ from CL mice are similar to that from WT mice.

### WBMCs from CL Mice Showed Improved Reconstitution Abilities during Serial Transplantation

**Figure 4:**
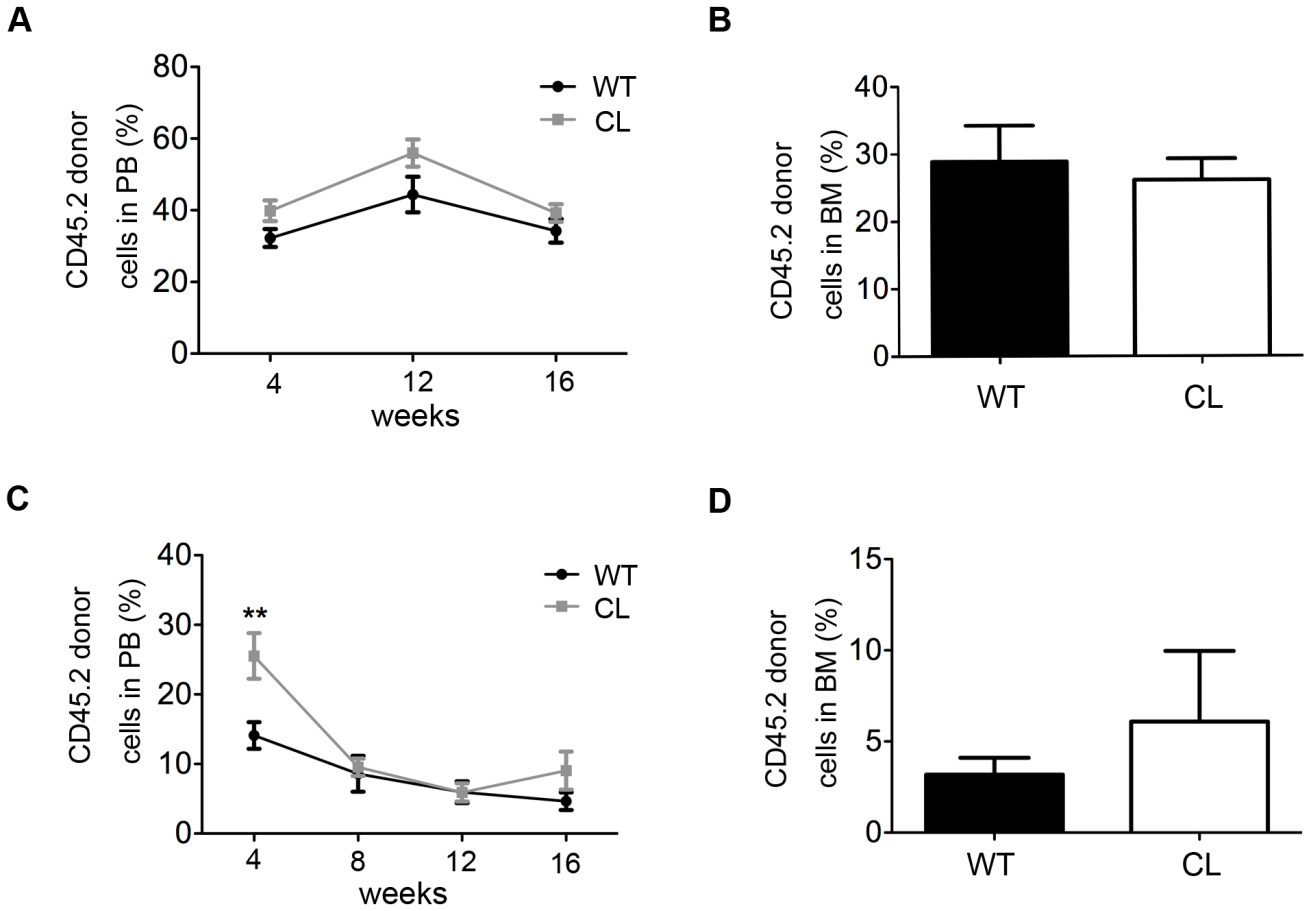
Improved reconstitution of CL WBMCs during serial transplantation. 1**^st^** transplantation: 5×10^5^ WBMCs from WT or CL mice plus 5×10^5^ CD45.1 WBMCs were transplanted into lethally irradiated (9.5 Gy) recipients (B6.SJL, CD45.1). (**A–B**) The percentage of CD45.2 donor cell in PB and BM at 4-week interval after transplantation. Data shown are mean ± SEM (n = 5–8). 2^nd^ transplantation: sorted 5×10^5^ CD45.2 cells from 1^st^ recipients of WT or CL mice, plus 5×10^5^ CD45.1 WBMCs were transplanted into lethally irradiated (9.5 Gy) recipients (B6.SJL, CD45.1). (**C–D**) The percentage of CD45.2 donor cell in PB and BM was analyzed every 4 weeks after transplantation. Data shown are mean ± SEM (**P<0.01, n = 3–4).

To determine whether low CPR expression affects the self-renewal ability of HSCs in CL mice, we performed serial transplantation with CL or WT mouse bone marrow cells. 5×10^5^ CD45.2 whole bone marrow cells (WBMC) of CL or WT mice plus 5×10^5^ CD45.1 WBMCs were transplanted into lethally irradiated (9.5 Gy) recipients (CD45.1). At 16 weeks post-transplantation, PB and BM cells were obtained and analyzed by flow cytometry. The repopulation capacity of WBMCs did not differ between CL and WT mice during the 1^st^ and 2^nd^ transplantations (Figure 4A–D). For the third transplantation, 2×10^5^ WBMCs from 2^nd^ transplantation recipients (CL or WT mice) were used. The repopulation capability of donor cells was evaluated after 8w of 3^rd^ transplantation. In reference to previously published papers such as by David T. Scadden and others [15], we set the threshold of 1% of engrafted CD45.2^+^ cells in PB as the standard of positive reconstitution. The reconstitution ratio was greater for WBMCs from CL mice (8 in 11 mice vs. 1 in 5 for the WT; P<0.05; Table 1). These results suggested that WBMCs from CL mice may have improved reconstitution abilities.

**Table 1:** Reconstitution upon third WBMCs transplantation.

	Reconstitution	No reconstitution	Total
WT recipient	1	4	5
CL recipient	8	3	11
Total	9	7	16

Third transplantation: 2×10^5^ CD45.2 cells sorted from 2^nd^ recipient WT or CL mice, plus 2×10^5^ CD45.1 WBMCs were transplanted into lethally irradiated (9.5 Gy) recipients (B6. SJL, CD45.1). Flow cytometry analysis of PB was performed after 8 weeks of transplantation. If the number of engrafted CD45.2^+^ cells in PB is greater than 1%, it was considered as positive reconstitution. Data were analyzed by χ^2^, P<0.05.

### WBMCs from WT Exhibit Decreased Lymphoid Differentiation Ratio and Increased Myeloid Differentiation Ratio in CL Recipients

**Figure 5:**
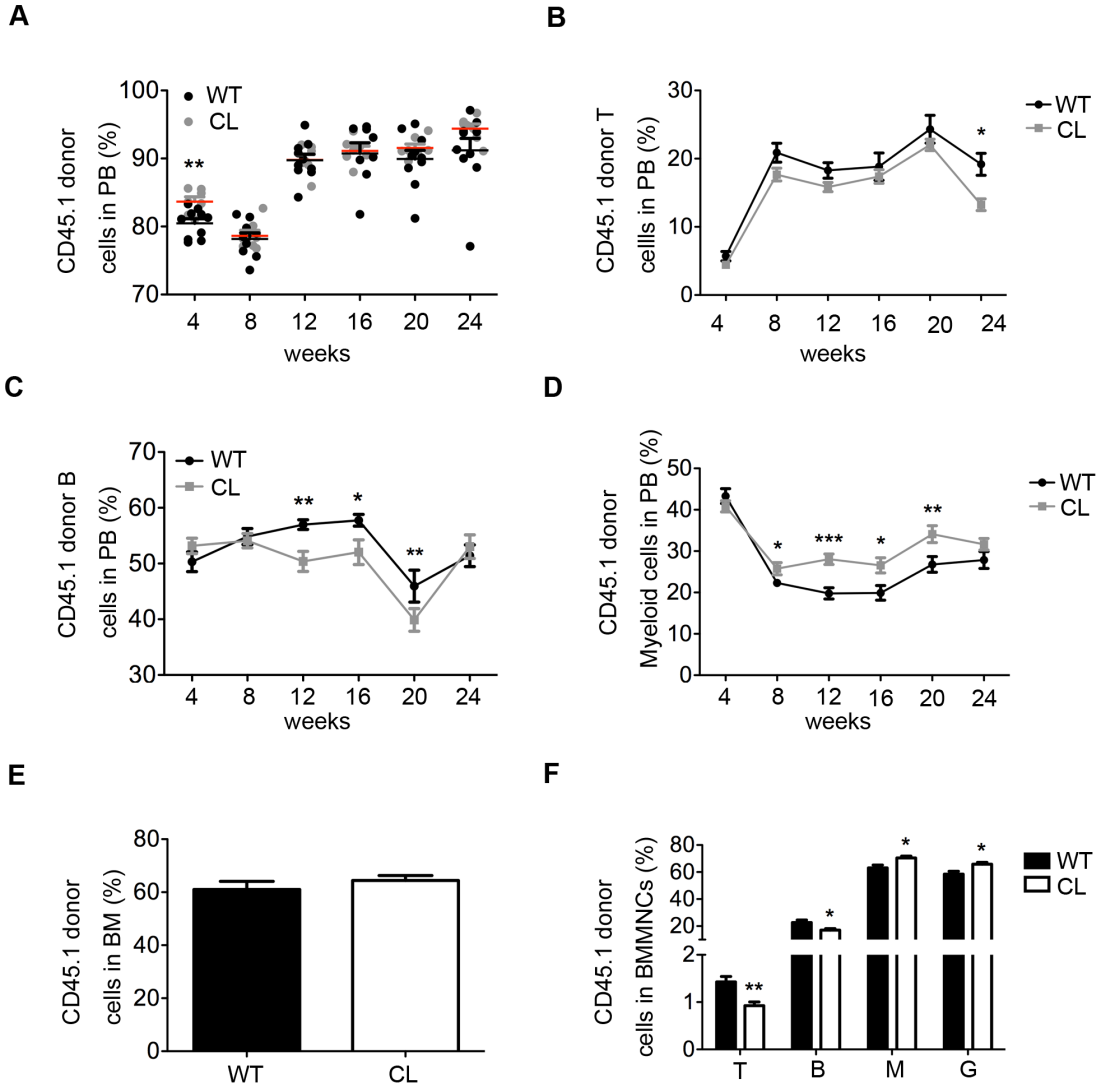
Reduced lymphoid differentiation ratio and increased myeloid differentiation ratio of WT WBMCs in CL recipients. 1×10^6^ CD45.1 WBMCs were transplanted into lethally irradiated (9.5 Gy) WT or CL mice. (**A**) Donor cell percentage in PB was analyzed by flow cytometry every 4 weeks. Data shown are mean ± SEM (n = 7–10). (**B–D**) The ratio of T lymphocyte, B lymphocyte and myeloid cell of donor cells in PB was analyzed by flow cytometry every 4 weeks. Data shown are mean ± SEM (*P<0.05, **P<0.01, ***P<0.001, n = 7–10). (**E**) The CD45.1 donor cell percentage in BM by flow cytometry at 24 weeks after transplantation. Data shown are mean ± SEM (n = 7–10). (**F**) T lymphocyte, B lymphocyte, myeloid cell and granulocyte percentage of CD45.1 donor cells in BM, analyzed by flow cytometry at 24 weeks after transplantation Data shown are mean ± SEM (*P<0.05, **P<0.01, n = 7–10).

We then performed recipient transplantation experiment in order to assess the effect of low CPR expression microenvironment on hematopoiesis. 1×10^6^ WBMCs from CD45.1 were transplanted into lethally irradiated WT and CL CD45.2 mice, respectively. Flow cytometry analysis showed comparable reconstitution capacity of WBMCs (from either PB or BM) after transplantation in CL vs WT mice (Figure 5A and E). LKS^+^ (HSCs) and LKS^−^ (HPCs) sub-populations from CD45.1 donor cells were then isolated from transplanted WT or CL mice for flow cytometry. The percentage of LKS^+/−^ donor cells was also similar between WT and CL mice (Figure S4), suggesting a minimal impact of the low CPR bone marrow microenvironment on transplanted WT HSCs/HPCs. Interestingly, lower T lymphocyte and B lymphocyte differentiation ratio was observed in PB and BM of CL recipient mice (Figure 5B, C and F) while myeloid cell differentiation ratio was higher in CL mice when compared with WT mice (Figure 5 D and F). These results indicated that HSCs might have increased ability for myeloid differentiation but decreased ability for lymphoid differentiation under the low CPR microenvironment.

### Hematopoietic Stem/progenitor Cells from CL Mice Maintain Normal Cell Cycle and Apoptosis Rate

**Figure 6:**
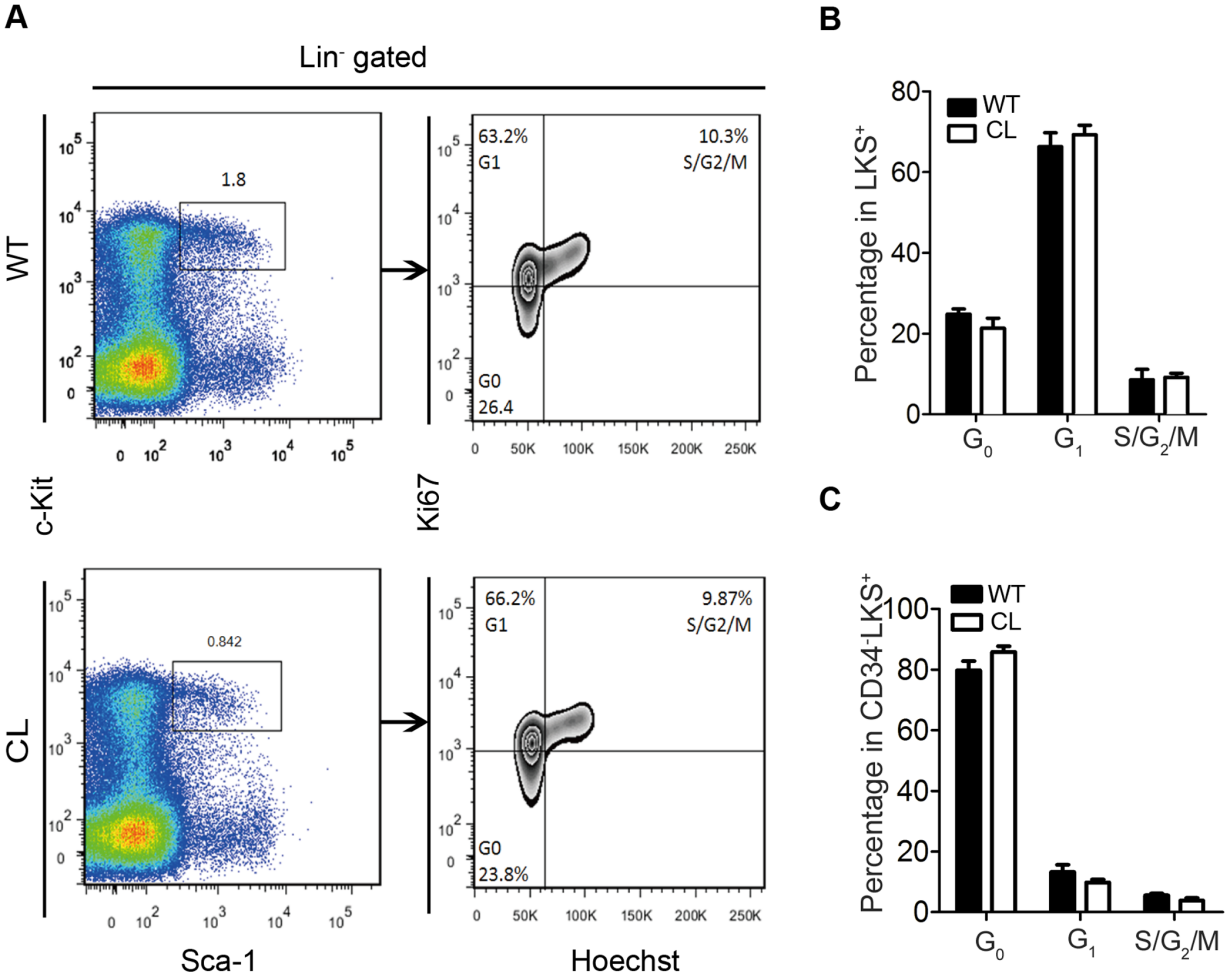
No alteration of cell cycle in hematopoietic stem/progenitor cells of CL mice. (**A–C**) Cell cycle status of LKS^+^ and CD34^−^LKS^+^ from WT vs. CL mice. (**A**) Results are representatives of flow cytometry data in figures. (**B–C**) Data shown are quantification of mean cell cycle status ± SEM (n = 3–4) in LSK^+^ (B) and CD34^−^LKS^+^ (C).

The quiescence of HSCs is essential for protecting the self-renewal ability of HSCs. To examine the cell cycle status in CL mice, we compared the cell cycle status of LKS^+^ and CD34^−^LKS^+^ cells in CL and WT mice with the markers for Ki67 and Hoechst33342 by flow cytometry. Cell cycle (G_0_, G_1_ vs. S/G_2_/M phases) of LKS^+^ and CD34^−^LKS^+^ cells did not differ between CL and WT mice (Figure 6A–C). Furthermore, apoptosis rate of LKS^+^ or LKS^−^ cells also did not differ between CL and WT mice (Figure S5).

## Discussion

HSC quiescence is required for their self-renewal and multi-potent differentiation capabilities, and is dependent on its residing microenvironment, including metabolic status. Using the low CPR expression CL mouse model, we showed that low CPR expression in HSCs can enhance the long-term repopulation efficiency of HSCs.

CPR is an obligated electron donor for all microsomal P450s responsible for metabolizing many important endogenous compounds. Deletion of the Cpr gene in mouse embryos leads to severe inhibition for vasculogenesis and hematopoiesis and causes embryonic lethality [12], [16], possibly due to the disruption of the homeostasis of endogenous substances (such as retinoic acids) metabolized by P450. Due to embryonic lethality of whole-body Cpr gene deletion, the knockout mouse model is not suitable to study the roles of CPR/P450 enzyme system in hematopoiesis in adult mice. The CL mouse, used in this study, having globally suppressed CPR expression (but not entirely absent), provides a unique model for investigating the *in vivo* roles of CPR/P450 enzyme system in hematopoiesis.

The current study showed that HSCs in CL mice have enhanced long-term repopulation ability despite of the lower number of HSCs presented in the mice. It is believed that the level of ROS in bone marrow is important for HSCs since high level of ROS could induce DNA damage and stimulate cell senescence in HSCs [1]. Conversely, low level of ROS could enhance the reconstitution ability of HSCs [6]. Thus, suppression of CPR expression in bone marrow might decrease and change the dynamic of ROS metabolism in the bone marrow cells and its microenviroment in CL mice although our results showed that the total ROS levels in BM, LKS^+^ and LKS^−^ populations of CL mice remained unchanged in comparison with that of WT mice. Accordingly, we speculate that CPR/P450 enzyme system is not a major source for ROS production for maintaining ROS levels in CL mice.

Cell cycle is regulated by cyclins, CDKs (cyclin dependent kinases), and CKIs (CDK inhibitors). CKIs are heavily involved in the regulation of HSCs. For example, HSCs of p18^−/−^ mice display enhanced self-renewal abilities [17]. The proliferation of HSCs from p21^−/−^ mice is increased despite of increased vulnerability to HSC exhaustion [18]. The p27^−/−^ mice show increased HPC proliferation but no change in HSC’s proliferation, cell cycle, and self-renewal ability [19]. CKI can also participate in the regulation of HSCs independent of cell cycle. To explore the possible mechanisms of this enhanced long-term repopulation ability of HSCs in CL mice, we investigated the possible changes of these parameters. However, no changes in cell cycle and apoptosis status were observed in hematopoietic stem/progenitor cells in CL mice although the expression levels of some CKIs were altered in CL mice (data not shown), possibly caused by a reduction of CPR/P450 expression in CL mice. Further studies on the links between P450 and CKIs are needed. Interestingly, we did not detect any obvious difference of the impact between CL and WT microenvironment when WT BM cells were transplanted onto irradiated CL mice except for the observation that the differentiation of myeloid lineage cells are slightly favored over lymphoid lineage cells. It will be interesting to revisit this issue in the CPR conditional knockout mouse models.

CPR/P450 enzyme system is responsible for the synthesis and degradation of many substances, including sex steroids, cholesterol, and retinoic acids. In the CL mice, the blood levels of testosterone and progesterone are markedly increased, whereas the blood levels of cholesterol are significantly decreased [14]. Sex hormones are negative regulators of lymphopoiesis [20]. For example, SSA (sex steroid ablation) enhances T cell recovery in mice following cyclophosphamide treatment [21], [22]. Immune system regeneration could enhance allogeneic or autologous hematopoietic stem cell transplantation by transient sex steroid blockade [23]. In our study, CL mice have elevated serum testosterone and progesterone levels which may cause decreased lymphoid differentiation and increased myeloid differentiation we observed (Fig. 5) [20]. Cholesterol also plays multiple roles in HSCs. Cholesterol efflux prevents HSPC mobilization and extra-medullary hematopoiesis [24] but does not affect the mobilization of hematopoietic stem cells [25]. CL mice have low circulating cholesterol level [26] although the potential impact on HSC repopulation remains to be studied.

In summary, our current study demonstrated for the first time that suppression of CPR/P450 enzyme system enhances the repopulation efficiency of HSCs and a microenvironment with low CPR expression levels favors the differentiation of myeloid over lymphoid lineage cells. Further studies are underway to dissect the molecular mechanisms for these intriguing findings.

## Supporting Information

Figure S1 **No changes in blood count and cellularity in hematopoietic organs in CL mice.** (**A**) The WBC, RBC and PLT cell count in PB of WT vs. CL mice. Data shown are mean ± SEM (n = 3–4). (**B**) The mononuclear cell number of the spleen (after lysing the red blood cells) and thymus in WT vs. CL mice. Data shown are mean ± SEM (n = 4). (**C**) The cellularity of BM from CL mice vs. WT. Data shown are mean ± SEM (n = 3–6).
(TIF)

Figure S2 **Normal bone marrow cell morphology and colony forming efficiency in CL mice.** (**A**) WT and CL bone marrow cells stained with Wright-Giemsa. (**B**) *In vitro* colony formation capacity of BMMNCs from WT vs. CL mice. 1×10^4^ BMMNCs from WT or CL mice were cultured with M3434 medium for 14 days. Data shown are mean ± SEM of colony number (n = 4).
(TIFF)

Figure S3 **No alteration in differentiation abilities of HSCs from CL mice** ***in vivo*** **.** 2.5×10^3^ LKS^+^ cells from WT or CL mice plus 5×10^5^ CD45.1 WBMCs were transplanted into lethally irradiated (9.5 Gy) recipients (B6.SJL, CD45.1). (**A–C**) The T lymphocyte, B lymphocyte and myeloid cell percentage in CD45.2 WT or CL mice donor cells in PB were detected every 4 weeks respectively. Data shown are mean ± SEM (n = 14–16). (**D**) T lymphocyte, B lymphocyte, myeloid cell and granulocyte percentage in CD45.2 donor cell in BM at 24 weeks after transplantation. Data shown are mean ± SEM (n = 5).
(TIF)

Figure S4 **Minimal impact of CL bone marrow on transplanted WT HSCs/HPCs.** 1×10^6^ CD45.1 WBMCs were transplanted into lethally irradiated (9.5 Gy) WT or CL mice. The LKS sub-population percentage of CD45.1 donor cells in BM was analyzed at 24 weeks after transplantation. Data shown are mean ± SEM (n = 7–10).
(TIF)

Figure S5 **No alteration of apoptosis in hematopoietic stem/progenitor cells of CL mice.** The apoptosis status in hematopoietic stem/progenitor cells from WT vs CL mice. Data shown are mean ± SEM (n = 4).
(TIFF)
