# Supplementary figures and images for: Suppression of Cytochrome P450 Reductase Enhances Long-Term Hematopoietic Stem Cell Repopulation Efficiency in Mice
Source: PLoS One. 2013 Jul 26;8(7):e69913. doi: 10.1371/journal.pone.0069913 (PMC3724780; doi:10.1371/journal.pone.0069913)

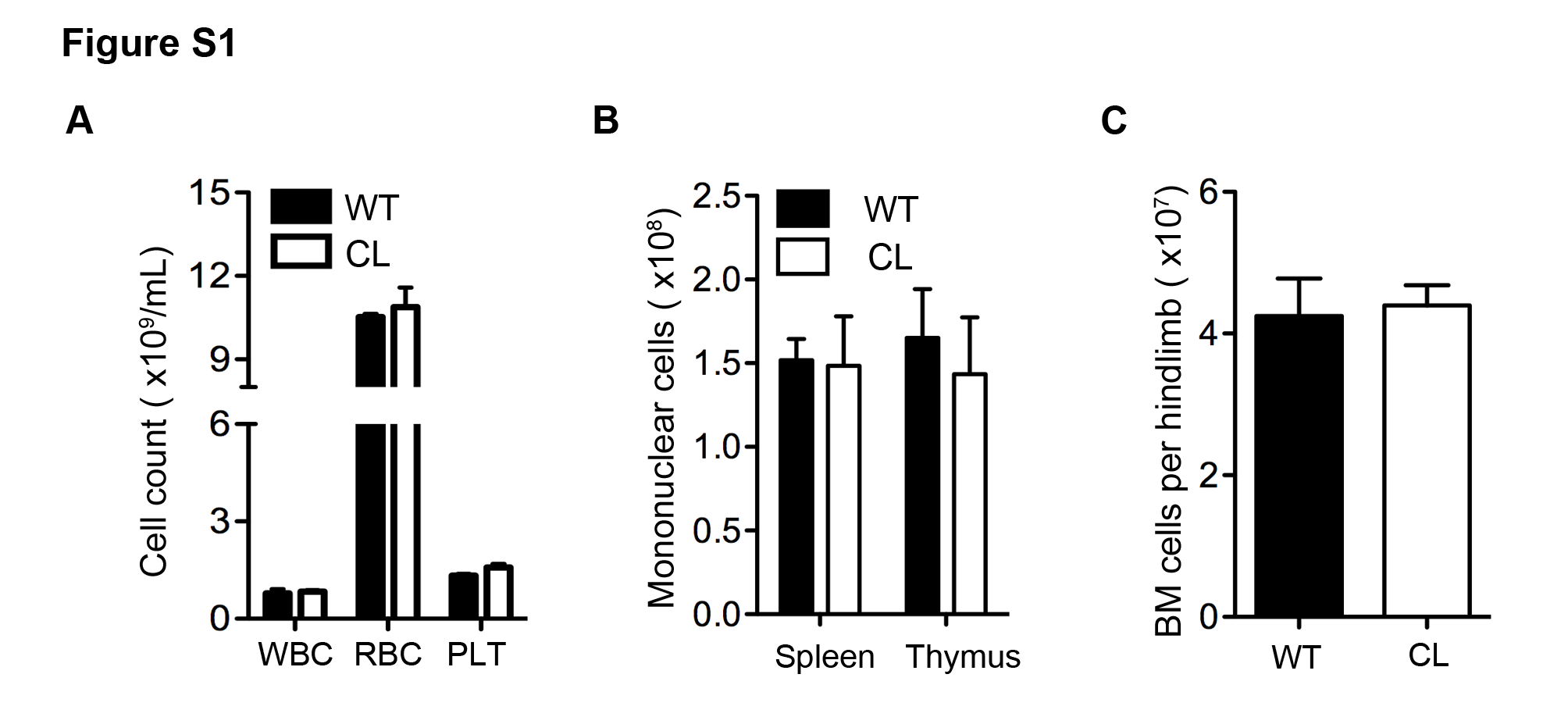

Supplement: Figure S1 — No changes in blood count and cellularity in hematopoietic organs in CL mice. (A) The WBC, RBC and PLT cell count in PB of WT vs. CL mice. Data shown are mean ± SEM (n = 3–4). (B) The mononuclear cell number of the spleen (after lysing the red blood cells) and thymus in WT vs. CL mice. Data shown are mean ± SEM (n = 4). (C) The cellularity of BM from CL mice vs. WT. Data shown are mean ± SEM (n = 3–6). (TIF) [file pone.0069913.s001.tif]

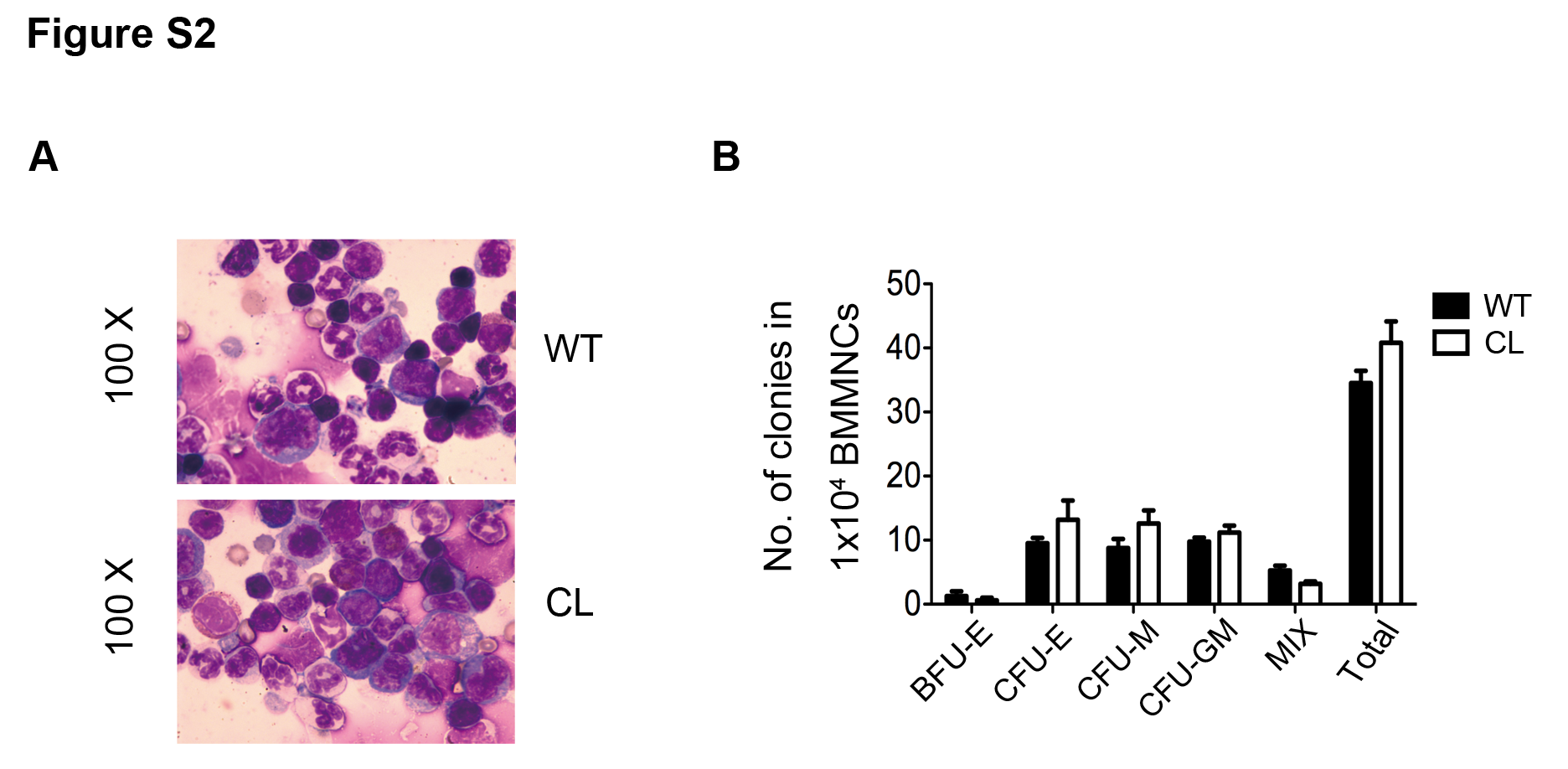

Supplement: Figure S2 — Normal bone marrow cell morphology and colony forming efficiency in CL mice. (A) WT and CL bone marrow cells stained with Wright-Giemsa. (B) In vitro colony formation capacity of BMMNCs from WT vs. CL mice. 1×10^4^ BMMNCs from WT or CL mice were cultured with M3434 medium for 14 days. Data shown are mean ± SEM of colony number (n = 4). (TIFF) [file pone.0069913.s002.tiff]

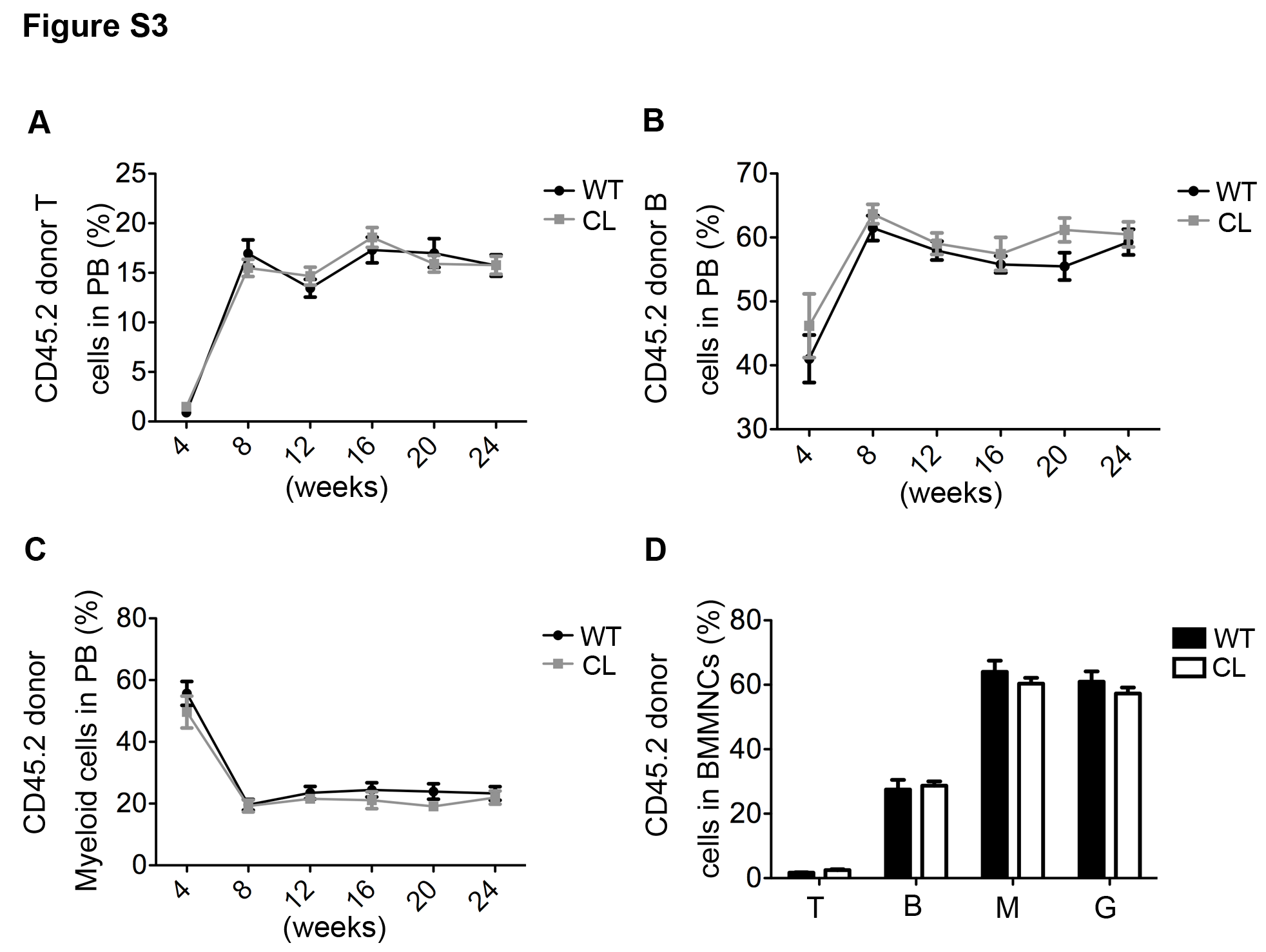

Supplement: Figure S3 — No alteration in differentiation abilities of HSCs from CL mice in vivo . 2.5×10^3^ LKS+ cells from WT or CL mice plus 5×10^5^ CD45.1 WBMCs were transplanted into lethally irradiated (9.5 Gy) recipients (B6.SJL, CD45.1). (A–C) The T lymphocyte, B lymphocyte and myeloid cell percentage in CD45.2 WT or CL mice donor cells in PB were detected every 4 weeks respectively. Data shown are mean ± SEM (n = 14–16). (D) T lymphocyte, B lymphocyte, myeloid cell and granulocyte percentage in CD45.2 donor cell in BM at 24 weeks after transplantation. Data shown are mean ± SEM (n = 5). (TIF) [file pone.0069913.s003.tif]

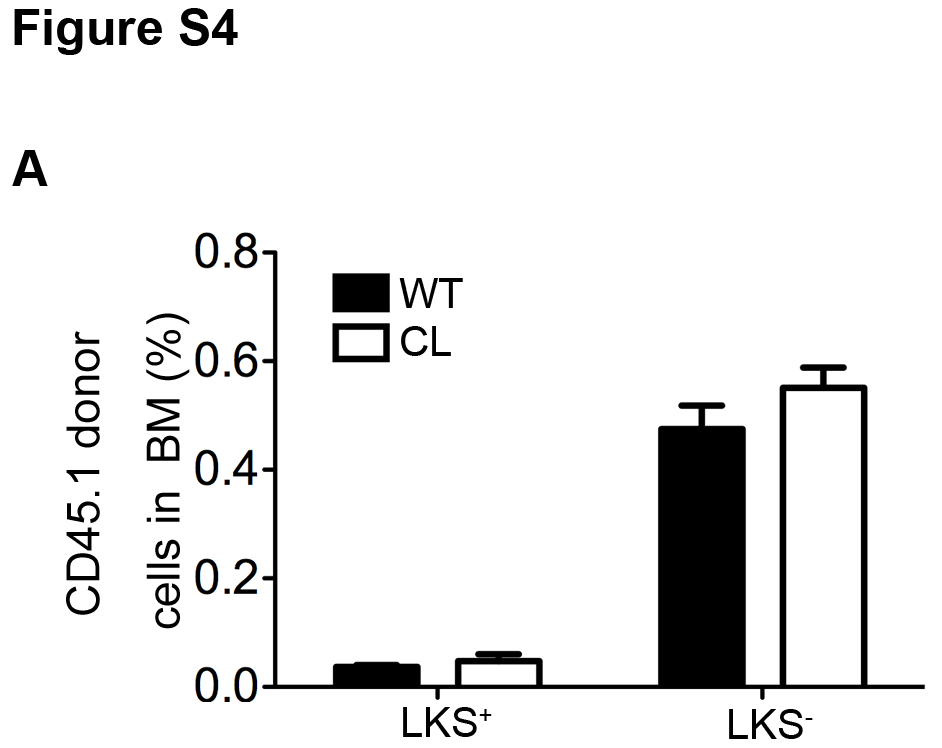

Supplement: Figure S4 — Minimal impact of CL bone marrow on transplanted WT HSCs/HPCs. 1×10^6^ CD45.1 WBMCs were transplanted into lethally irradiated (9.5 Gy) WT or CL mice. The LKS sub-population percentage of CD45.1 donor cells in BM was analyzed at 24 weeks after transplantation. Data shown are mean ± SEM (n = 7–10). (TIF) [file pone.0069913.s004.tif]

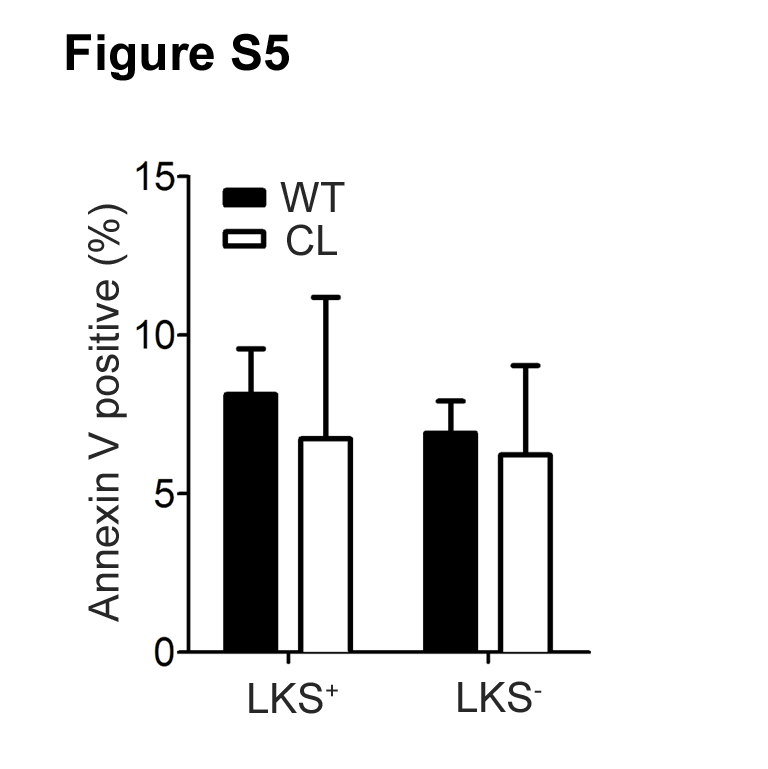

Supplement: Figure S5 — No alteration of apoptosis in hematopoietic stem/progenitor cells of CL mice. The apoptosis status in hematopoietic stem/progenitor cells from WT vs CL mice. Data shown are mean ± SEM (n = 4). (TIFF) [file pone.0069913.s005.tiff]
